# The Simplified Acute Physiology Score III Is Superior to the Simplified Acute Physiology Score II and Acute Physiology and Chronic Health Evaluation II in Predicting Surgical and ICU Mortality in the “Oldest Old”

**DOI:** 10.1155/2014/934852

**Published:** 2014-02-17

**Authors:** Aftab Haq, Sachin Patil, Alexis Lanteri Parcells, Ronald S. Chamberlain

**Affiliations:** ^1^Saint George's University School of Medicine, West Indies, Grenada; ^2^Department of Surgery, Saint Barnabas Medical Center, Livingston, NJ, USA; ^3^Department of Surgery, University of Medicine and Dentistry of New Jersey (UMDNJ), 94 Old Short Hills Road Livingston, Newark, NJ 07039, USA

## Abstract

Elderly patients in the USA account for 26–50% of all intensive care unit (ICU) admissions. The applicability of validated ICU scoring systems to predict outcomes in the “Oldest Old” is poorly documented. We evaluated the utility of three commonly used ICU scoring systems (SAPS II, SAPS III, and APACHE II) to predict clinical outcomes in patients > 90 years. 1,189 surgical procedures performed upon 951 patients > 90 years (between 2000 and 2010) were analyzed. SAPS II, SAPS III, and Acute APACHE II were calculated for all patients admitted to the SICU. Differences between survivors and nonsurvivors were analyzed using the Student's *t*-test and binary logistic regression analysis. A receiver operating characteristic (ROC) curve was constructed for each scoring system studied. The area under the ROC curve (aROC) for the SAPS III was 0.81 at a cut-off value of 57, whereas the aROC for SAPS II was 0.75 at a cut-off score of 44 and the aROC for APACHE II was 0.74 at a cut-off score of 13. The SAPS III ROC curve for prediction of hospital mortality exhibited the greatest sensitivity (84%) and specificity (66%) with a score of 57 for the “Oldest Old” population.

## 1. Introduction

Life expectancy has increased substantially in the past half century due to significant advances in healthcare prevention alongside improvements in diagnosis and treatment approaches. As a result, the most rapidly growing segment of the US population is the elderly, defined as individuals older than 65 years [1]. The “Oldest Old” in the population are those over 85, which currently represents 2% of the US census—a figure, that is, expected to increase over 200% by 2050 [1]. These changing demographics have already had a dramatic effect on ICU admissions, with mean age of patients admitted and total number of ICU admissions increasing faster than healthcare resources can keep pace [2]. Information derivable from validated ICU scales will likely play an increasingly important role in guiding physician decision making and may facilitate evidence-based rationing of limited healthcare resources in the future.

To date, numerous studies have documented the negative impact of advanced age on ICU outcomes [2–7]. Although older age is clearly associated with increased mortality, other age-related factors signifying severity of illness have been shown to be better at predicting ICU outcomes in elderly patients than age alone [8, 9]. These factors include the admitting diagnosis [8, 10–13], comorbidities [14–18], and the functional status of the patient prior to ICU admission [19–22]. Commonly used ICU prognostic scoring models include the Simplified Acute Physiologic Score II (SAPS II), Acute Physiology and Chronic Health Evaluation II (APACHE II), and the newly developed SAPS III. These scoring systems incorporate physiologic parameters, co-morbidities, admitting diagnoses, Glasgow coma scales, and age to provide a numerical score that can in turn predict ICU mortality.

Sakr et al. compared the utility of SAPS III against APACHE II and SAPS II in 1851 surgical ICU patients (mean age of 62 years). They noted that in-hospital mortality was substantially greater in patients with higher SAPS III score, and that a score greater than 80 was associated with a 70% mortality rate whereas a score less than 40 was associated with a less than 3% mortality. The authors concluded that the SAPS II and SAPS III predict mortality better than the APACHE II model in elderly patients [23].

Healthcare advancements in recent decades have permitted more elective surgeries in patients with very advanced age. However, suitable literature documenting the ICU outcomes of this age group is lacking. This study sought to evaluate the utility of the SAPS II, SAPS III, and APACHE II scoring systems in nonagenarians (>90 years) admitted to the surgical ICU.

## 2. Materials and Methods

A retrospective review of all nonagenarians admitted to Saint Barnabas Medical Center (SBMC) in Livingston, NJ, over a 10-year period (between 2000 and 2010) was performed. 951 unique nonagenarian patients were admitted who underwent 1189 surgical procedures. 117 (9.8%) of those patients were admitted to the Surgical Intensive Care Unit (SICU) postoperatively. Pertinent data was collected using a standard data collection sheet after approval from the institutional review board (IRB: 10–25). Data abstracted included age, gender, comorbidities, procedure type, ASA status, operative time, hospital length of stay, ICU length of stay, ICU admission, and outcome. SAPS II, SAPS III, and APACHE II scores and predicted mortality were calculated by retrospective chart review for 89 patients (28 were excluded due to insufficient chart data). Two study populations were grouped into a mortality group and a survivor group. The mortality group included all patients who died within the SICU and the survivor group consisted of all patients who were discharged. Receiver Operator Characteristic (ROC) Curves were plotted to determine the sensitivity and specificity in the aforementioned ICU scoring models to predict in-hospital mortality in this population.

The outcomes of ICU patients, especially mortality, depend on several factors. Based on these factors, several severity scoring systems have been developed. The severity sores usually comprise two parts: the score itself (higher number indicates higher severity) and a probability model (an equation giving the probability of in-hospital death). The most commonly used severity scoring systems include APACHE II, SAPS II, and SAPS III. The APACHE II was developed by a panel of experts based on their personal opinion whereas SAPS II and SAPS III were developed by prospective multi-institutional studies. Differences between the abovementioned scoring systems are shown in Table 1.

## 3. Results

See Table 2.

### 3.1. Age and Sex

The mean overall patient age was 93.2 years (91–100); the mean age among male patients was 92.9 years, while the mean age among female patients was 93.4 years. The M : F ratio was 1 : 1.02. On SICU admission, the survivor group's mean age was 93.2, whereas the mortality group's mean age was 92.8 years, *P* < 0.5.

### 3.2. Length of Stay and Discharge Status

The mean stay for all patients admitted to the SICU was 6 ± 8 days and the mean hospital stay was 16.6 ± 10 days. The majority of discharged patients were sent to a nursing facility (*N* = 30; 33.7%) or home without assisted living (*N* = 29; 32.6%). The remainder of the patients were discharged to a cancer center (*N* = 8; 9%), or rehabilitation center (*N* = 4; 4.4%), while 14 patients (15.7%) suffered mortality.

### 3.3. Comorbidities

The co-morbidities most prevalent in our study population were cardiac diseases. These include congestive heart failure (CHF) in 38.2% (*N* = 34) patients, hypertension in 34.8% (*N* = 31) patients, atrial fibrillation in 29.2% (*N* = 26) patients, and coronary artery disease in 21.3% (*N* = 19) patients.

### 3.4. Anesthesia

The preoperative American Society of Anesthesiologists (ASA) score was available for 64 patients, and the mean ASA score was 3.31 (range: 2–5). The mean ASA score for male patients was 3.33 (range: 2–5) compared to 3.15 (range: 2–4) in female patients, *P* = 0.4. Twenty-eight percent (*N* = 18) of patients had an ASA score of two, 56.2% (*N* = 36) had an ASA score of three, 12.5% (*N* = 8) had an ASA score of four while only 3.1% (*N* = 2) of patients had an ASA score of five. General anesthesia was provided to 74.2% of the patients (*N* = 66), cardiac anesthesia to 10.1% (*N* = 9), and regional anesthesia to 7.9% (*N* = 7). The remaining 7.9% (*N* = 7) of cases were performed under Monitored Anesthesia Care (MAC).

### 3.5. Surgery

Among the surgical procedures performed 38.2% (*N* = 34) had general surgery, orthopedic surgery 13.5% (*N* = 12), cardiac surgery 10.1% (*N* = 9), urologic surgery 9% (*N* = 8), neurosurgery 7.9% (*N* = 7), vascular surgery 6.7% (*N* = 6), and 14.6% (*N* = 13) of patients had invasive procedures (endoscopy, cystoscopy, and biopsy). The mean operative time was 152 ± 112 minutes.

### 3.6. SAPS II, SAPS III, and APACHE II Scores

The overall mortality in the studied group was 15.7% (14 of 89). The mean SAPS II, score (predicted mortality) for patients who died was 57.4 ± 20.0 (55.2% ± 29.7%) compared to 41.7 ± 14.9 (30.5% ± 23.7%) for survivors, *P* < 0.001. The mean SAPS III score (predicted mortality), for patients who died, was 74.6 ± 14.2 (60.7% ± 22.1%) compared to 57.8 ± 14.5 (32.4% ± 23.6%) for survivors, *P* < 0.001. The mean APACHE II score (predicted mortality), for patients who died, was 23.1 ± 8.7 (46.4% ± 26.4%) compared to 16.0 ± 7.0 (26.8% ± 19.1%) for survivors, *P* < 0.001 (Table 3).

**Figure 1:**
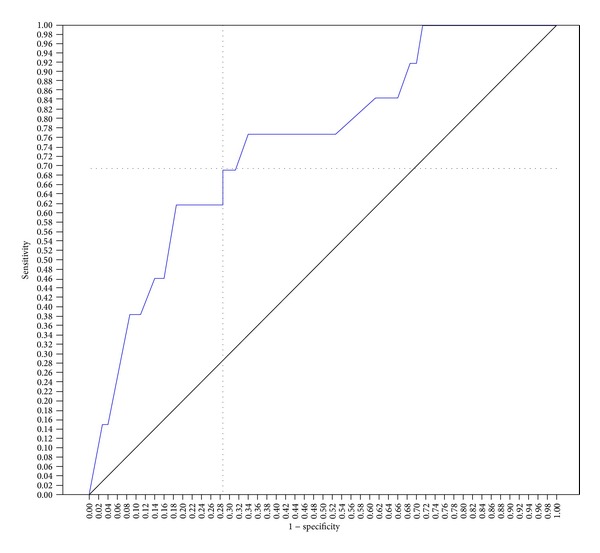
SAPS II ROC curve for prediction of hospital mortality. The score of 44 showed better sensitivity (77%) and specificity (65%) for hospital mortality, with an area under the curve of 0.75 (area = 0.5; *P* < 0.004, 95% CI; 0.60–0.89).

**Figure 2:**
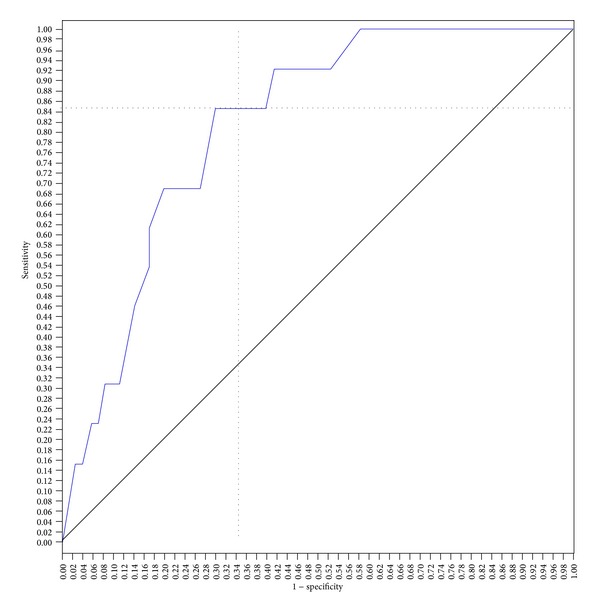
SAPS 3 ROC curve for prediction of hospital mortality. The score of 57 showed better sensitivity (84%) and specificity (66%) for hospital mortality, with an area under the curve of 0.81 (area = 0.5; *P* < 0.0001, 95% CI; 0.70–0.92).

**Figure 3:**
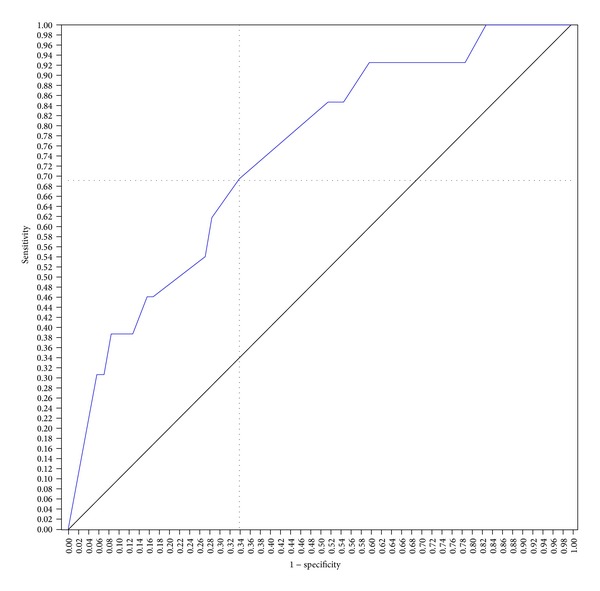
APACHE 2 ROC curve for prediction of hospital mortality. The score of 13 showed better sensitivity (69%) and specificity (66%) for hospital mortality, with an area under the curve of 0.74 (area = 0.5; *P* < 0.006, 95% CI; 0.59–0.88).

Using a cut-off score of 44, the SAPS II score predicted hospital mortality with a sensitivity of 77% and a specificity of 65%, with an area under the ROC curve (aROC) of 0.75 (95% CI; 0.60–0.89, *P* < 0.004). With a cut-off score of 57, SAPS III score predicted hospital mortality with a sensitivity of 84% and a specificity of 66%, with an aROC of 0.81 (95% CI; 0.70–0.92, *P* < 0.0001). With a cut-off score of 13, the APACHE II score predicted hospital mortality with a sensitivity of 69% and specificity of 66%, with an aROC of 0.74 (95% CI; 0.59–0.88, *P* < 0.006). The area under the curve for the SAPS III ROC (aROC) curve was 0.81 compared to 0.75 and 0.74 for SAPS II score and APACHE II score, respectively, indicating that the SAPS III score best predicted hospital mortality in this study population (Figures 1, 2, and 3).

## 4. Discussion

Elderly patients represent nearly 50% of all ICU admissions and account for 60% of ICU days [5]. As the Baby Boomers generation approaches retirement age (65 years), the gap between overall resources and patient needs will rapidly expand exponentially. Advances in healthcare prevention, diagnostics, and treatment modalities have markedly expanded lifespan beyond predicted expectations and a growing body of surgical literature documents improved surgical outcome in the “Oldest Old” is further proof of this fact. Between 1990 and 2000, the total number of abdominal aorta aneurysm (AAA) repairs, coronary artery bypass Graft (CABG), carotid endarterectomy (CEA), colon resections, and lung resections performed on patients older than 80 has increased dramatically with an acceptable 30-day mortality rate of 8.4% [24]. Less commonly, feasible surgical outcomes in nonagenarians and centenarians have also recently been documented [24]. Despite these isolated results, little known questions remain about how we identify “Oldest Old” patients who are likely to do well following surgery versus those who will not. Current economic times have made us all acutely aware that healthcare resources are not intangible, and given the fact that many studies suggest that we spend up to 50% of a patient's entire healthcare expenditure in the last 6 months of their life, viable solutions as who is among likely to benefit from different interventions is vital to future decision making. At present, a number of validated survey systems have been published that can provide some guidance as to how we should ration limited healthcare resources such as surgical intervention and ICU admission in catering younger patient population but whether this applies to “Oldest Old” is unknown.

The SAPS II and APACHE II prognostic models are the most commonly used scoring systems for critically ill patients admitted to the ICU [25–27]. In 2005, the SAPS III model was proposed and differs primarily from the former two models in the fact that data is collected within the first hour following ICU admission rather than within 24 hours [28, 29]. Nearly half of the predictive power of the SAPS III score is based on information available prior to ICU admission, making it a potential tool for ICU triage as well. Scoring systems that utilize data derived 24 hours after ICU admission obviously have no utility for ICU screening as that data reflects the ICU care provided. Several studies have looked at the utility of SAPS II and APACHE II in surgical patients, but only two studies have described the utility of SAPS III in surgical patients. Furthermore, until now there are no studies that analyze the utility of SAPS III in surgical patients of very advanced age.

All three scoring systems have the ability to predict survivorship (known as discrimination) and to evaluate the predicted mortality against the observed mortality (known as calibration) [30]. This study demonstrates that the SAPS III has slightly better discrimination than the SAPS II and APACHE II in surgical ICU patients over 90-year old. These results are consistent with the limited published literature available on the SAPS III scores evidence in surgical patients. Silva et al. studied 1,310 surgical patients with a mean age of 67.1 and found that a SAPS III score of 57 yielded an aROC of 0.86 [31]. Unlike the current study, they did not evaluate the disparity between the various available scoring systems. Sakr et al. evaluated 1851 surgical patients with a mean age of 62 and found that the SAPS III had an aROC of 0.84, which was higher than both the SAPS II and APACHE II of 0.83 and 0.80, respectively [23].

The SAPS III score was developed with data from 303 ICUs and 16,784 patients worldwide [28, 29]. Though comprehensive, the SAPS III data was not representative of all types of patient populations since it was developed using a general ICU population pool. As a result, external validation remains essential before applying this score to any specific patient population, including surgical patients and the elderly. Although our outcomes are similar to those of Sakr et al., our study group consisted only of patients over 90 years of age with the vast majority of patients having underwent general surgery. Further, it is difficult to draw specific conclusions about percent increased risk in elderly patients for major or minor procedures from this study set; other studies have clearly documented an increased risk for any invasive procedure in the frail and debilitated elderly patient.

Although the result of this study serves as an external validation for the SAPS III score in nonagenarian surgical ICU patients, this is a retrospective pilot study and as such there are several limitations to the study design. The power of the study is limited by the small number of patients studied and the fact that the surgical case mix was predominantly in the field of general surgery for gastrointestinal diseases.

In conclusion, the SAPS III is a valuable tool for predicting mortality in surgical ICU patients older than 90 years of age. Given the ease of SAPS III calculations, it may also be a useful tool for ICU triage of “Oldest Old” surgical patients and may assist the physician in making difficult decisions regarding the rationing of healthcare resources and the aggressiveness of initial ICU care.

## Figures and Tables

**Table 1:** Differences between APACHE II, SAPS II, and SAPS III severity scoring systems.

	APACHE II	SAPS II	SAPS III
Variables	Rectal Temp, MAP, HR, RR, Aa gradient/Po_2_, pH/HCO_3_, Na, K, creatinine, Hct, WBC, GCS, Age, chronic diagnosis	Age, type of admission, temp, SBP, HR, GCS, UOP, WBC, BUN, K, Na, HCO_3_, bilirubin, Pao_2_/Fio_2_, AIDS, metastatic carcinoma, hematologic malignancy	Age, LOS before ICUA, Intrahospital location (OR, ER, other ICU, other), comorbidities (cancer therapy, cancer, hematologic cancer, AIDS, Chronic HF (NYHA IV), Cirrhosis), Vasoactive drugs before ICUA, ICU admission (planned, unplanned), Reason for Admission (cardiovascular, hepatic, digestive, neurologic), Surgical Status at ICUA (scheduled surgery, emergency surgery, no surgery), site of surgery (transplant, trauma, cardiac surgery, neurosurgery), acute Infection at ICUA (nosocomial, respiratory), GCS, highest Total Bilirubin, highest body temperature, highest creatinine, highest HR, lowest WBC count, lowest pH, lowest platelet, lowest SBP, MV or CPAP PaO_2_/FiO_2_
Data collection	Within 24 hours of admission to ICU	Within 24 hours of admission to ICU	Within 1 hour of admission to ICU
Major limitation	Not helpful to stratify outcome prediction based on primary diagnosis	May be less accurate for noncardiovascular diseases	

Temp: Temperature, MAP: mean arterial pressure, HR: heart rate, RR: respiratory Rate, Aa: alveolar-arterial, Po_2_: partial pressure of oxygen; pH: hydrogen ion concentration, HCO_3_: bicarbonate concentration, Na: sodium ion concentration, K: potassium ion concentration, Hct: hematocrit, WBC: white blood cell count, GCS: Glasgow Coma Scale, Temp: temperature, SBP: systolic blood pressure, UOP: urine output, BUN: blood urea nitrogen, Fio_2_: fraction of inspired oxygen, AIDS: Acquired Immune Deficiency Syndrome, LOS: length of stay, ICUA: intensive care unit admission, HF: heart failure, NYHA: New York Heart Association, MV: minute ventilation, CPAP: continuous positive pressure ventilation.

**Table 2:** Demographics and clinical characteristics for 89 nonagenarians admitted to the surgical ICU between 2000 and 2010.

	Overall	Mortality group	Survivor group
Total patients, *N* (%)	89	14 (16)	75 (84)
Mean age, years (range)	93.2 (91–100)	92.8 (91–96)	93.2 (91–100)
Male : female	1 : 1.02	1 : 1.1	1 : 1.06
Comorbidities, *N* (%)			
CHF	34 (38)	7 (50)	27 (36)
Hypertension	31 (35)	3 (21)	28 (37)
Atrial fibrillation	26 (29)	4 (29)	22 (29)
CAD	19 (21)	2 (14)	17 (23)
ASA grade, *N* (%)			
I	0	0	0
II	18 (25)	0 (0.0)	18 (24)
III	36 (49)	3 (21)	33 (44)
IV	18 (25)	2 (14)	16 (21)
V	2 (3)	2 (14)	0
Type of anesthesia, *N* (%)			
General anesthesia	66 (74)	13 (93)	53 (71)
Cardiac anesthesia	9 (10)	1 (7)	8 (11)
Regional anesthesia	7 (8)	0	7 (9)
MAC	7 (8)	0	7 (9)
Types of procedures, *N* (%)			
General surgery	34 (38)	10 (71)	24 (32)
Orthopedic surgery	12 (14)	0	12 (16)
Cardiac surgery	9 (10)	1 (7)	8 (11)
Urologic surgery	8 (9)	0	8 (11)
Neurosurgery	7 (8)	1 (7)	6 (8)
Vascular surgery	6 (7)	2 (14)	4 (5)
Invasive procedures*	13 (15)	0	13 (17)
Mean operative time, min ± SD	152 ± 112	152.3 ± 148.0	138.8 ± 103.0
Mean ICU stay, days ± SD	6 ± 8	12.0 ± 6.5	5.0 ± 0.8
Mean length of hospital stay, days ± SD	16.6 ± 15	15.5 ± 14.1	17.4 ± 15.3
Discharge status, *N* (%)			
Nursing facility	30 (34)	—	30 (40)
Home without assisted living	29 (33)	—	29 (39)
Cancer center	8 (9)	—	8 (11)
Rehabilitation center	4 (4)	—	4 (5)

*N*: number of patients, CHF: congestive cardiac failure, CAD: coronary artery disease, ASA: American Society of Anesthesiologists, MAC: managed anesthesia care, min: minutes, SD: standard deviation, ICU: intensive care unit.

*Invasive procedures included endoscopy, cystoscopy, and biopsy.

**Table 3:** Comparison of ICU mortality prediction models based on mean score and area under the receiver operator curve for 89 nonagenarians admitted to surgical ICU between 2000 and 2010.

Prediction models	Mortality group scores (mean ± SD) *N* = 14 (16%)	Survivor group scores (mean ± SD) *N* = 75 (84%)	Area under ROC curve (95% CI)	*P* value
SAPS II	57.4 ± 20.0	41.7 ± 14.9	0.75 (0.60, 0.89)	*P* < 0.02
SAPS III	74.7 ± 14.2	57.8 ± 14.5	0.81 (0.70, 0.92)	*P* < 0.001
APACHE II	23.1 ± 8.7	16.0 ± 7.0	0.74 (0.59, 0.88)	*P* < 0.02

SD: Standard deviation, ROC: receiver operator curve, CI: Confidence Interval, *N*: number of patients, SAPS: standardized Acute Physiology Score, APACHE: Acute Physiology and Chronic Health Evaluation.
